# Seizure Forecasting Using a Novel Sub-Scalp Ultra-Long Term EEG Monitoring System

**DOI:** 10.3389/fneur.2021.713794

**Published:** 2021-08-23

**Authors:** Rachel E. Stirling, Matias I. Maturana, Philippa J. Karoly, Ewan S. Nurse, Kate McCutcheon, David B. Grayden, Steven G. Ringo, John M. Heasman, Rohan J. Hoare, Alan Lai, Wendyl D'Souza, Udaya Seneviratne, Linda Seiderer, Karen J. McLean, Kristian J. Bulluss, Michael Murphy, Benjamin H. Brinkmann, Mark P. Richardson, Dean R. Freestone, Mark J. Cook

**Affiliations:** ^1^Seer Medical Pty Ltd, Melbourne, VIC, Australia; ^2^Department of Biomedical Engineering, The University of Melbourne, Melbourne, VIC, Australia; ^3^Department of Medicine at St. Vincent's Hospital Melbourne, The University of Melbourne, Fitzroy, VIC, Australia; ^4^Epi-Minder Pty. Ltd., Melbourne, VIC, Australia; ^5^Cochlear Limited, Sydney, NSW, Australia; ^6^Department of Neuroscience, St. Vincent's Hospital Melbourne, Fitzroy, VIC, Australia; ^7^Department of Neuroscience, Monash Medical Centre, Melbourne, VIC, Australia; ^8^Department of Medicine, School of Clinical Sciences at Monash Health, Monash University, Melbourne, VIC, Australia; ^9^Bioelectronics Neurophysiology and Engineering Lab, Department of Neurology, Mayo Clinic, Rochester, MN, United States; ^10^School of Neuroscience, Institute of Psychiatry, Psychology and Neuroscience, King's College London, London, United Kingdom

**Keywords:** seizure, seizure cycles, seizure forecasting, epilepsy, implantable device, sub scalp

## Abstract

Accurate identification of seizure activity, both clinical and subclinical, has important implications in the management of epilepsy. Accurate recognition of seizure activity is essential for diagnostic, management and forecasting purposes, but patient-reported seizures have been shown to be unreliable. Earlier work has revealed accurate capture of electrographic seizures and forecasting is possible with an implantable intracranial device, but less invasive electroencephalography (EEG) recording systems would be optimal. Here, we present preliminary results of seizure detection and forecasting with a minimally invasive sub-scalp device that continuously records EEG. Five participants with refractory epilepsy who experience at least two clinically identifiable seizures monthly have been implanted with sub-scalp devices (Minder^®^), providing two channels of data from both hemispheres of the brain. Data is continuously captured via a behind-the-ear system, which also powers the device, and transferred wirelessly to a mobile phone, from where it is accessible remotely via cloud storage. EEG recordings from the sub-scalp device were compared to data recorded from a conventional system during a 1-week ambulatory video-EEG monitoring session. Suspect epileptiform activity (EA) was detected using machine learning algorithms and reviewed by trained neurophysiologists. Seizure forecasting was demonstrated retrospectively by utilizing cycles in EA and previous seizure times. The procedures and devices were well-tolerated and no significant complications have been reported. Seizures were accurately identified on the sub-scalp system, as visually confirmed by periods of concurrent conventional scalp EEG recordings. The data acquired also allowed seizure forecasting to be successfully undertaken. The area under the receiver operating characteristic curve (AUC score) achieved (0.88), which is comparable to the best score in recent, state-of-the-art forecasting work using intracranial EEG.

## Introduction

For people with epilepsy, an estimation of total seizure burden is fundamental to clinical management as well as for the evaluation of new therapies, such as drugs or devices. For over a century, clinicians have relied on their patients' reports of their seizure frequency, “that it may be taken as an index of the severity of the epileptic condition” (1). Although the rate of clinical seizures influences an individual's perception of disease severity, the physiological basis for this remains ambiguous (2, 3). Indeed, the number of clinical seizures is not representative of (nor closely correlated with) the total seizure burden (4). Rates of subclinical epileptiform activity seen on electroencephalography (EEG) are typically orders of magnitude higher than clinical seizures. These subclinical events may impact cognition (5, 6) and quality of life, and are important in epilepsy diagnosis and treatment, particularly for syndromes that are characterized by stereotypical discharges. Interictal epileptiform activity is also relevant for surgical planning (7) and forecasting seizure likelihood (8). Therefore, capturing both clinical and subclinical events, and interictal epileptiform activity, is important for the clinical management of epilepsy. Henceforth, we define Epileptiform Activity (EA) as interictal and ictal epileptic activity, comprising interictal discharges and electrographic events (clinical and subclinical). Often we specify “interictal EA,” which refers to interictal epileptiform discharges only.

The easiest and most common method of capturing clinical seizure events is through patient self-reporting. Unfortunately, the accuracy of self-reported events is unreliable (9, 10). In addition to unawareness of subclinical events, patients are often unaware or forgetful of their clinical seizures, and may also report other non-epileptic symptoms as seizures. As there have been no real alternatives, seizure diaries (both paper and electronic) are used almost exclusively to manage patients, and regulatory authorities assess new treatments primarily on evidence from diaries (11). It is possible that the unreliability of self-reporting has impeded progress in the development of anti-seizure medications (12). In addition to inaccurate records of seizure frequency, people with epilepsy and caregivers typically cannot provide an objective assessment of the time of seizure onset, seizure duration or seizure type (11). This detailed information about seizures is important for patient management, particularly with regard to medication titration and safety. For this reason, capturing EEG correlates of seizures remains the reference standard in clinical epilepsy management.

Short-term (up to 10 days) inpatient video-EEG assessment can be used to assess treatment efficacy, for surgical planning, and has been proposed as an objective metric for randomized controlled trials. However, short-term monitoring has major limitations. The spatiotemporal organization of interictal EA, including epileptiform spikes and high frequency oscillations (HFOs), changes over long time scales (months to years), so short-term capture of interictal EA is unreliable (13, 14). In addition, seizure rates show high natural variability and require long-term recording to identify clinically relevant improvements (15, 16). Short-term monitoring is particularly inadequate for people with lower seizure frequencies and cannot detect multiday cycles of interictal EA that occur in most individuals (17, 18).

Ultra-long term monitoring is required for better diagnosis, management and treatment of epilepsy, including seizure forecasting. Currently, scalp EEG is not suitable for ultra-long term monitoring due to limited data quality and the need for external electrode maintenance (19). Invasive intracranial systems, such as the RNS System (NeuroPace) and the Percept PC (Medtronic), are available but are built for neurostimulation, do not store sufficient data and are too invasive for diagnostic applications (19). Alternatively, sub-scalp EEG systems are minimally-invasive tools that may address the need for objective ultra-long term EEG recordings (19, 20), allowing for personalized and accurate epilepsy management.

Our earlier work with an implantable intracranial device (4) demonstrated that continuous EEG permitted characterization of EEG features (21, 22), epileptic activity (18, 23) and sleep (24, 25), and enabled successful seizure forecasting (26–28). As similar data could be acquired from a less invasive (sub-scalp) EEG recording system, we have developed a minimally invasive device that is inserted into a sub-scalp location to continuously record EEG. This work reports on the feasibility of the system to detect interictal EA and seizures in five subjects. Therefore, the primary aim of this manuscript was to report on the preliminary results of interictal EA and seizure detection using the sub-scalp device, and to qualitatively compare these recordings to reference-standard 7-day ambulatory video-EEG monitoring. As a secondary aim, we also present a case study to illustrate the potential for seizure forecasting using sub-scalp EEG. The case study provides a proof-of-concept on how cycles can be derived from event detections in the EEG and how these cycles can be used to forecast epileptic seizures. The presented forecasting method builds on previous work in seizure cycles (20, 29–31) and interictal EA cycles (17, 18, 32).

## Materials and Methods

### Patient Selection and Criteria

Data used in this work were acquired during a registered trial (ACTRN 12619001587190). Subjects participating in the Minder^®^ sub-scalp system (Table 1) trial were 18-75 years of age at the time of implantation, had an established clinical diagnosis of epilepsy (33) with a minimum of two clinically identifiable epileptic seizure events per month, and otherwise were medically and neurologically stable as defined by their clinician. All participants had EEG profiles that were consistent with epilepsy diagnosis, and had prior neuroimaging. Subjects were excluded if they had a neurostimulation implant device for epilepsy or another condition, or had any other condition that may impact the study outcome or safety of the device.

**Table 1:** Participant demographics.

**Participant**	**Gender**	**Age (years)**	**Epilepsy type**	**Epilepsy etiology**	**Seizure onset localization**	**Seizure types**	**Antiepileptic medications**
1	F	49	Multifocal	Periventricular nodular heterotopia	Multifocal	Focal impaired Aware-ness	Lacosamide sodium Valproate, Pregabalin, Brivaracetam
2	M	60	Focal	Cortical dysplasia	Right temporo-parietal junction	Focal impaired Aware-ness	Carbamazepine, Lacosamide
4	F	44	Focal	Hypothalamic hamartoma	Hypo-thalamus	Focal impaired Aware-ness	Carbamazepine, Brivaracetam, Phenobarbital, Clonazepam
5	F	47	Focal	Non-lesional temporal lobe epilepsy	Right temporal lobe	Focal impaired aware-ness	Lamotrigine
6	M	45	General-ized	Genetic generalized epilepsy	Generalized	Absence, general-ized tonic-clonic	Sodium valproate, levetiracetam, lamotrigine, zonisamide

All participants wore the sub-scalp system for at least 8 months during both wake and sleep. Subjects were also expected to maintain a seizure diary, if necessary with the assistance of a caregiver, and attend regular study appointments. All participants gave written, informed consent and the study protocol was approved by St Vincent's Hospital Melbourne Human Research Ethics Committee (HREC 063/15).

### Implantable System

**Figure 1:**
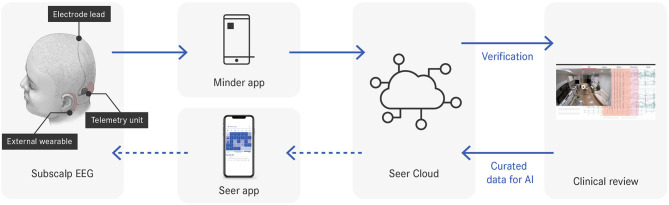
Data flow throughout the system. Dotted arrows indicate system components under development.

The Minder^®^ sub-scalp system (Epi-Minder Pty Ltd) is an investigational device comprising an implanted device, which communicates with an external wearable unit, a mobile phone and a secure cloud (Figure 1). The implanted device is positioned under the scalp, with a small burrhole to recess the telemetry device and includes an electrode array that is passed superiorly with two contacts located over each parietal bone. The electrodes record differential EEG signals across two contacts at 250 Hz, which are captured by the telemetry unit. The telemetry unit communicates with an external behind-the-ear (BTE) processor via an inductive radio frequency (RF) link, which allows data and power transfer between the external wearable device and the implant. The BTE processor communicates with a mobile phone via Bluetooth. The mobile phone application (Minder app) facilitates the transfer of EEG data from the implant to the phone, and ultimately to a secure cloud for processing. The Minder app also captures audio and accelerometry data from the phone and stores it together with the EEG data in the Seer Cloud (Seer Medical Pty Ltd). Data captured by the implanted device is reviewed and curated on the Seer Cloud platform. Curated events are used for training a machine learning algorithm that detects EA and whose output is used for seizure forecasting. In future, seizure forecasting will be delivered to patients through the Seer App.

### Surgical Procedure and Follow Up Assessments

The device is implanted under general anesthetic in a specific position for the implanted receiver located in the mastoid bone. The electrode array is passed subcutaneously and over the pericranium, posterior to the vertex and over the parietal regions. The location of the sub-scalp electrode was chosen to optimize EA detection rates (modeling from scalp data indicated this produced the highest yield of event capture) and minimize artifact from nearby temporalis muscles. The surgical procedure for implantation of the Minder implant housing and coil was modeled closely on that used for commercial cochlear implants. Regular check-ups (every 2–6 weeks) were conducted in person and participants communicated with study doctors and coordinators between in-person visits. In addition, 7-day scalp EEG was performed at weeks 4 and 24 after implantation. The scalp EEG recordings consisted of a standard 10–20 electrode placement with an additional four scalp electrodes placed as close as practically feasible to the underlying implanted sub-scalp electrodes. The purpose of the scalp EEG assessment was to compare the sub-scalp EEG signal to the scalp EEG, particularly during seizures and interictal EA, and as well activities including sleep, and potential sources of artifact. Subjects were asked to keep their seizure diaries during monitoring so that the three modalities of seizure detection (sub-scalp implant, scalp EEG and seizure diary) could be compared.

### Epileptic Activity Detection

For both sub-scalp EEG and ambulatory EEG, the detection of EA was aided by a machine learning algorithm designed to detect relevant events in the EEG (34, 35). The algorithm was designed to label suspect EA with high sensitivity to ensure that all interictal and ictal events were detected.

EEG recordings and suspect events highlighted by the algorithm were accessed from the cloud through an online portal and reviewed by expert neurophysiologists, who marked interictal EA and seizures. Interictal EA marked by the neurophysiologists consisted of typical epileptiform EEG activity such as spike discharges. EEG seizures consisted of EEG activity substantially larger than background and lasting a minimum of 10 s. Seizure morphologies were first confirmed in each participant by observing correlated seizures in the ambulatory scalp video-EEG, however ambulatory scalp EEG and sub-scalp EEG were reviewed independently when compared qualitatively in this manuscript.

### Seizure Forecasting Case Study

In this case study, we demonstrate the potential for forecasting using sub-scalp EEG in participant 1. This participant was chosen because of their comparatively larger amount of reviewed data and high seizure count relative to the other participants.

This retrospective case study was designed using training and testing datasets. To train the forecasting algorithm, we utilized cycles in both machine-detected suspect events and manually confirmed electrographic seizures to forecast seizure likelihood per hour. During testing, the forecaster attempted to predict human confirmed electrographic seizures.

#### Data Pre-processing and Feature Extraction

Two features were incorporated into the forecaster: significant cycles based on rates of machine-detected events and significant cycles based on seizures. To compute event-based cycles, we used a similar approach to a previously published method for extracting rhythms of EA (17). Briefly, a Morlet wavelet transform was computed on the z-standardized hourly event rates to produce a global wavelet spectrum of power for each scale (cycle period). The cycle periods considered were every 1.2 h between 2.4 and 31.2 h, every 2.4 h between 33.6 and 48 h, every 4.8 h between 52.8 and 4 days and every 12 h between 5 days and up to a maximum period of a quarter of the recording duration. At least four cycle periods had to be present to confirm a cycle. Peaks in the wavelet spectrum were found by comparing neighboring values. Peaks above the global significance (99% confidence) level were determined to be significant EA cycle periods using a time-averaged significance test (36).

Once significant cycle periods were computed, event rates were filtered at each significant cycle period using a zero-order Butterworth bandpass filter. The bandpass filter used cut-off frequencies at ±33% of the cycle frequency [consistent with (17)]. These cut-off frequencies were chosen to account for phase shifts in the cycle over the recording time. To account for bandpass overlap in significant cycle frequencies, we introduced a sparsity criterion whereby only the strongest peak (greatest power in the wavelet spectrum) within any cycle's bandpass filter pass band was considered. The instantaneous phase of the cycle at each timepoint was then estimated using a Hilbert transform. Filtered cycles in event rates were used as features for the forecaster if seizures were significantly phase-locked to the cycle [*p* < 0.05, according to the omnibus/Hodges-Ajne test for circular uniformity (37)].

Cycles in seizure times were detected using a similar approach to our previous work (29, 38). We assessed the phase locking of seizure times to a range of possible cycles using both the Omnibus test (*p* < 0.05) and the synchronization index (SI ≥ 0.4) value to quantify phase locking. The SI value–a measure of the magnitude of the resultant vector–ranges from 0 to 1, where 0 represents a perfectly uniform circular distribution and 1 represents perfect alignment with respect to an underlying cycle (30). To account for multiple cycle periods meeting the criteria within close proximity, we used only the strongest cycle period (based on the highest SI value) within ±33% of any other cycle period.

All features were transformed from cyclical to linear features by normalizing the signals from 0 to 2π and computing the sine and cosine of the normalized signal.

#### Forecasting Algorithm

To forecast the likelihood of a seizure on an hourly basis, we used an ensemble machine learning algorithm that combined a random forest (RF) regressor and a logistic regression (LR) classifier. The output of the model was the final likelihood of a seizure (risk value), which was represented as a continuous value between 0 for no seizure and 1 for a guaranteed seizure within the next hour. Note that a likelihood was given every hour based on “clock hours” (e.g., 12 a.m., 1 a.m., etc) rather than just an arbitrary moving time window.

The RF regressor with the bootstrap aggregating technique was trained on all features. In the model, the number of decision trees was 80 and the minimum number of samples required to be at a leaf node was 15. From observation, these model parameters achieved the highest accuracy on the training dataset. Since seizures typically account for < 1% of daily life (4), the dataset is usually imbalanced, with non-seizure hours occurring far more frequently than seizure hours. RF models typically performed better on balanced datasets (39), so oversampling of seizure hours was undertaken before training the RF model. The output of the RF model was used as an input to the LR classifier. The LR classifier was trained on all features, including the output of the RF model. For simplicity, the default logistic regression model was used from Python's *sklearn* library. The output of the LR model was the final likelihood of a seizure (risk value) within the next hour.

Using the likelihood values, the forecaster classified hours as either low, medium or high risk. The medium and high risk cut-off thresholds were computed by optimizing (26):

(C1) time spent in low risk > time spent in medium risk > time spent in high risk;

(C2) seizures in high risk > seizures in medium risk > seizures in low risk;

If C1 or C2 could not be satisfied, the optimisation algorithm maximized the product of the time in low risk and the number of seizures in high risk (C3 and C4):

(C3) maximize the time spent in a low risk state;

(C4) maximize the number of seizures occurring in the high risk state.

Note that the likelihood is distinct from a traditional probability value where all outcomes sum to 1. This distinction is caused by oversampling the seizure class in the RF model, which generates synthetic seizure-hours such that the number of seizure hours is equal to the number of non-seizure hours. The result is that the likelihood values are higher than the true probability values.

#### Training and Testing Datasets

After preprocessing and feature extraction, the dataset was split into training and testing datasets. Initial algorithm training occurred using seizures captured over the first 14 days (15 seizures) but using cycles derived from the entire dataset. After the initial training, re-training occurred after each new seizure was observed. Re-training occurred on all past data, which recomputed the algorithm coefficients and risk thresholds for future predictions.

All analyses were executed in Python (version 3.7.9) using pandas (v1.2.0), numpy (v1.19.2), matplotlib (v3.3.2), datetime (v3.7.9), scipy (v1.5.2), pycwt (v0.3.0), sklearn (0.23.1), imblearn (v0.6.2) and pycircstat (v0.0.2) libraries.

## Results

The surgical procedure and devices were well tolerated. No significant complications have been reported in the five participants. Overnight use of the system was well tolerated and the BTE processor was worn either on the ear or attached to the clothing during sleep. The 7-day EEG recordings revealed interictal EA in all participants and seizures in four of the five participants (Table 2).

**Table 2:** Clinically relevant EEG events during the two 7-day EEG sessions.

**Participant**	**Interictal EA discharges**	**Seizures**	**Patient reported seizures (confirmed events)**
1	S1: 1476	S1: 27	S1: 7 (6)
	S2: 5981	S2: 17	S2: 1 (1)
2^*^	S1: 245	S1: 3	S1: 6 (0)
4	S1: 179	S1: 3	S1: 4 (3)
	S2: 52	S2: 0	S2: 0
5	S1: 519	S1: 0	S1: 3 (0)
	S2: 110	S2: 0	S2: 0
6	S1: 5783	S1: 5	S1: 1 (0)
	S2: 2084	S2: 0	S2: 0

*Confirmed events are seizures confirmed through clinical review*.

*S1 - 7-day monitoring at 4 weeks post-implant*.

*S2 - 7-day monitoring at 24 weeks post-implant*.

*: *Note participant 2 did not have monitoring at 24 weeks*.

**Figure 2:**
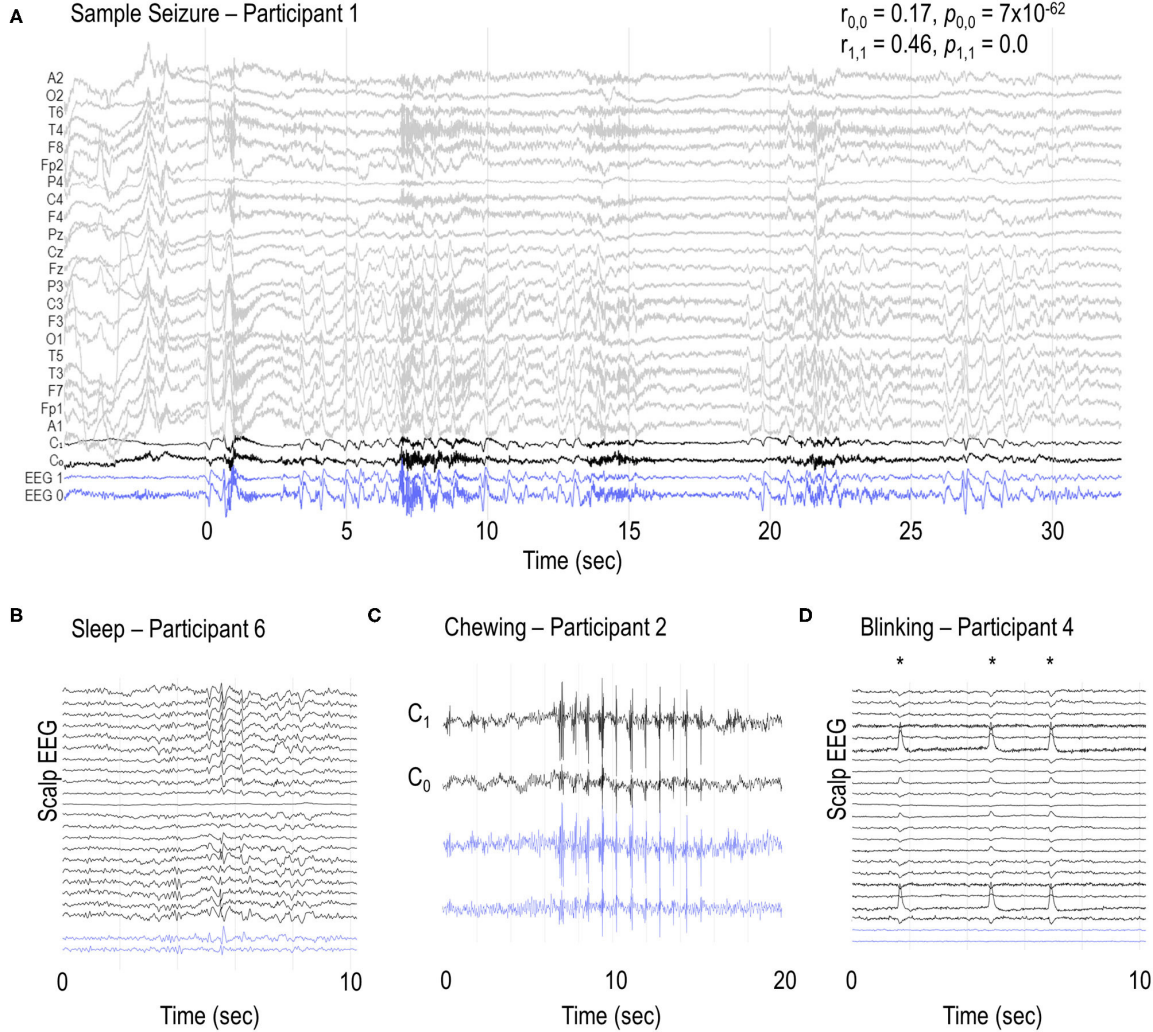
Sample EEG recordings. **(A)** Sample seizure in participant 1 and examples of **(B)** vertex waves and spindles during sleep, **(C)** chewing and **(D)** blinking artifacts (*) in participants 6, 2 and 4, respectively. C_0_ and C_1_ channels represent the bipolar recordings from the additional scalp electrodes placed over the sub-scalp electrodes. Blue traces (EEG 0 and EEG 1) represent the sub-scalp recordings. r_0,0_ and r_1,1_ represent the Pearson correlation coefficient between C_0_ and EEG 0, and C_1_ and EEG 1, respectively. *p*_0,0_ and *p*_1,1_ represent the respective p values. Ambulatory scalp EEG and sub-scalp EEG recordings were reviewed independently.

Seizures were identified on the sub-scalp system, as confirmed by periods of concurrent conventional scalp EEG recordings (Figure 2A). Many other neurological events and artifacts were also present in the sub-scalp recordings. In all participants, clear sleep-related transients were visible in the sub-scalp recordings (Figure 2B). Head scratching and muscular artifacts, such as chewing or jaw clenching artifacts, typically appeared very large across the sub-scalp recordings (Figure 2C), while other artifacts, such as blinking, were largely invisible (Figure 2D).

### Seizure Forecasting Case Study

**Figure 3:**
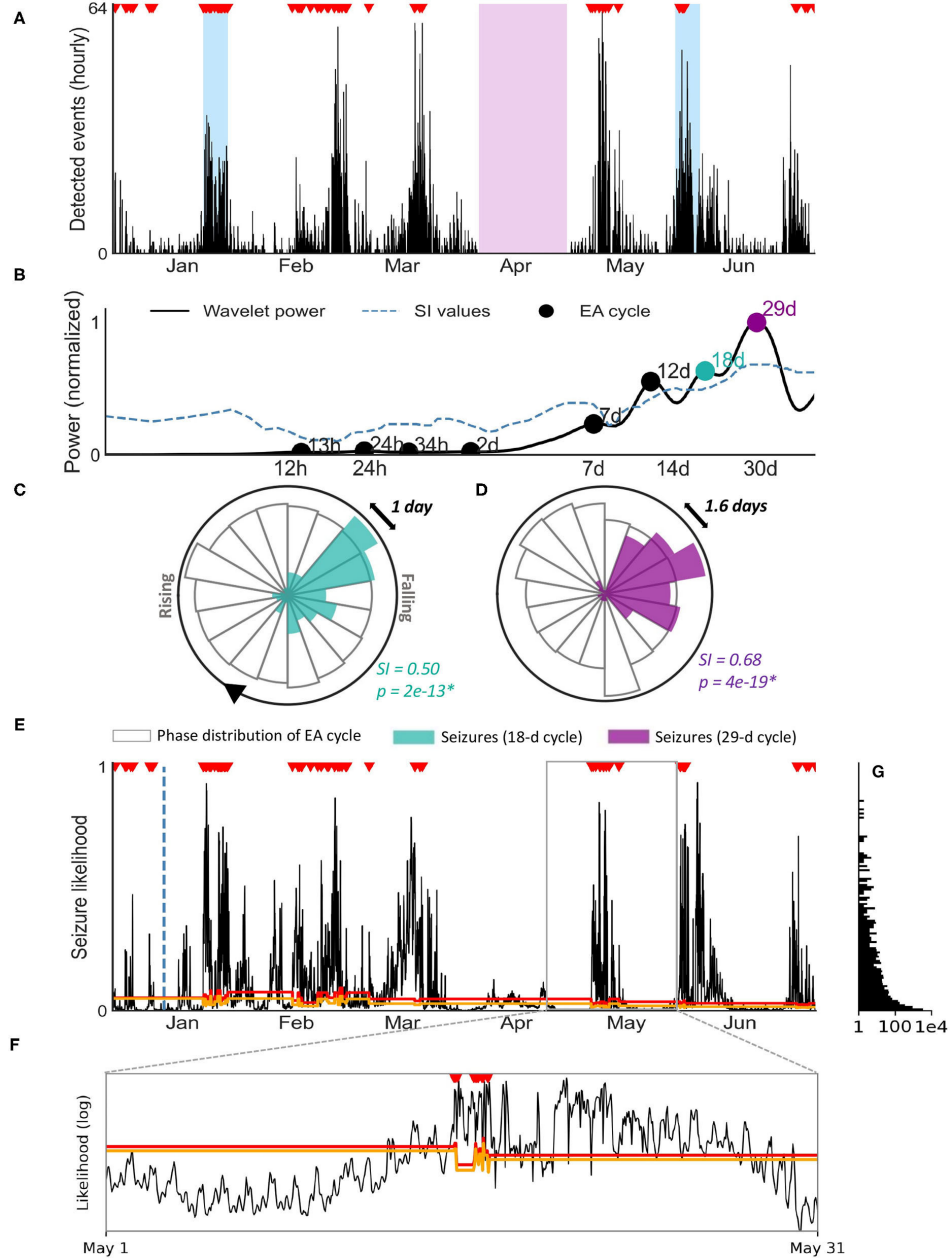
Seizure forecasting case study for patient 1. **(A)** Hourly rate of machine-learning detected events using the sub-scalp EEG device. Verified seizures (red markers) from manual review of sub-scalp EEG device are shown. The blue regions represent scalp video-EEG assessment periods and the purple region represents a period where the device was removed. **(B)** Cycle detection in EA using a Morlet wavelet approach. The wavelet spectrum is shown for a range of time periods (x-axis), with cycles reaching significance denoted by a black or colored marker. The two colored markers indicate the two cycles shown in the circular histograms **(C,D)**. **(C,D)** Circular histograms showing the phase distribution of event cycles (transparent bars) and seizure occurrence (colored bars). Seizures were strongly locked onto 18 day **(C)** and monthly cycles **(D)** of detected events. Seizures only occur in a narrow phase of the periodic activity suggesting a strong relationship between the cycle and seizures (*p* < 0.05 with Omnibus test and high SI values). **(E)** Hourly likelihood of seizures. The likelihood of seizures occurring within the next hour is given by the black line and seizures are shown by the red markers.The training cut off date (day 14) is indicated by the blue dotted line. The orange and red lines represent the medium and high risk thresholds, respectively. **(F)** Inset of **(E)**: hourly likelihood (x-axis, log scale) of seizures for the month of May, with seizures and threshold lines shown. **(G)** Frequency (x-axis, log scale) of each seizure likelihood value [y-axis, shared with **(E)**].

We conducted a proof-of-principle analysis of seizure forecasting for Participant 1. This participant had a total of 134 seizures over a 6 month period. Figure 3A shows the hourly rate of detected events from a machine learning algorithm (see Methods: epileptic activity detection) over the 6 month period. Shaded blue regions represent the two 7-day EEG sessions recorded at weeks 4 and 24. The purple region represents an extended period where data was not collected (device was removed). Note that the device was removed during this period to undertake a physical examination of the scalp for any changes to the skin before reapplication of the system, as part of our safety assessment process.

Figure 3A highlights the presence of multi day cycles (approximately monthly). The hourly event counts were used to identify significant periodic cycles ranging from 12 h to 40 days, as shown in the wavelet spectrum in Figure 3B. Confirmed seizures were only phased locked to some of these cycles (quantified by significant SI values). Two examples of seizure phase locking to cycles of hourly event count (18 days and 29 days) are shown (Figures 3C,D).

A practical forecaster minimizes the amount of time the forecaster displays a high risk warning while maximizing the number of seizures occurring during high risk. Alternatively, an opposite, suboptimal forecaster would always show high risk, achieving perfect predictive performance but of no utility to the end-user. Figure 3E demonstrates seizure likelihood over 6 months in participant 1, where risk levels have been optimized to be of highest utility to the user. The likelihood trace peaks in a cyclical manner, with seizures typically occurring close to the peaks (Figure 3F). The participant had 134 seizures during this period, 15 of which occurred in the first 14 days (initial training phase) and 119 of which occurred during the testing period.

The time spent in low, medium and high risk warnings and seizures that occured during these periods are given in Table 3 for the testing phase. The distribution of seizure likelihoods can also be visualized in Figure 3G. The forecast resulted in the participant spending 26% of time in a high risk state, 11% of time in a medium risk state and 63% of time in a low risk state. Of 119 testing seizures, 99 (83%) occurred during high risk, 8 (7%) occurred during medium risk and 12 (10%) occurred during low risk. The median time spent in the high risk state before a seizure occurred was 28 h. The Area Under the Receiver Operating Characteristic Curve (AUC score), which demonstrates how good the model is at distinguishing non-seizure hours from seizure hours during testing, was 0.88. These results underscore the feasibility of seizure forecasting using data from the sub-scalp EEG device.

**Table 3:** Forecast results based on electrographic seizures.

	**In high risk state**	**In medium risk state**	**In low risk state**
Seizures	99 (83%)	8 (7%)	12 (10%)
Time	26%	11%	63%

*Number of electrographic seizures occurring in and time spent in high, medium and low risk states during testing. Training was performed on electrographic seizures. AUC = 0.88*.

## Discussion

Here, we have successfully shown that a sub-scalp system can accurately record ultra-long term EEG (> 12 months) and detect focal seizure activity (Figure 2). The device was well tolerated in all five participants, with no serious adverse events to date. This suggests that continuous monitoring of EA chronically is possible with a minimally invasive and discrete device. The benefits of this are ubiquitous, not only for seizure forecasting, but also for medication management, anti-seizure medication trials and surgical planning. It is highly likely that accurate and objective quantification of seizures and interictal EA will become essential for future drug trials to provide more objective assessments of therapeutic benefit; sub-scalp EEG would be highly suitable for this purpose.

Our results demonstrate that sub-scalp devices record high quality neurological signals that are similar to scalp EEG. Sub-scalp recordings are also sensitive to other small neurological events such as sleep transients (Figure 2B). Lack of sleep and deviations from normal sleep patterns are known risk factors for seizures. Conversely, the treatment of seizures and seizures themselves can disrupt normal sleep patterns (40, 41). Sub-scalp devices provide an opportunity to investigate the complex relationship between sleep and seizures and can aid in patient management and seizure forecasting (39). The sub-scalp EEG was less noisy compared to scalp EEG. Sub-scalp EEG contained much less interference from electrical line noise (50 Hz in Australia) and was not affected by movement artifacts typically observed in scalp EEG due to the movement of wires. Sub-scalp devices are, however, susceptible to other noise and artifacts, such as muscle activity recorded by electromyography (EMG). While jaw EMG artifacts may obscure the underlying EEG activity (Figure 2C), it can also identify jaw activity that is a feature of the seizures. In contrast, blinking artifacts could not be seen in the sub-scalp recordings, most probably because of the parietal positioning of electrodes (Figure 2D).

The sub-scalp device can be used to continuously monitor interictal and ictal events, which may provide better understanding of the burden of disease. This information is also of importance for clinical trials of novel therapies and for routine patient management. Currently, clinical trials of novel therapies rely on patient seizure diaries, which are known to be unreliable in most people (11). The inconsistency of patient seizure diaries impacts the estimate of disease burden and distorts the estimated benefit of new therapies. Our case study demonstrates long-term fluctuations in detected events which were linked to seizures (Figure 3A).The detected events are likely to represent similar fluctuations in EA, which have been implicated in cognition and memory performance (5, 6). Understanding how EA changes over time is important for tailoring treatments that not only reduce seizures but ultimately improve quality of life.

The Minder^®^ sub-scalp system demonstrated utility in capturing seizure cycles. In the current work, there was clear rhythmicity in the detected events (Figure 3A), which is concordant with previous work with invasive EEG showing the prevalence of circadian and multiday cycles in interictal EA (17, 18) and seizures (20, 29, 31). Using a similar approach to previous work (20, 40), cycles were detected at circadian and multiday periodicities for one individual (Figure 3B), with 18-day and 29-day cycles in the detected events showing the strongest relationships with seizure timing (Figures 3C,D). Interestingly, multiday cyclesin this subject were stronger than the circadian rhythm. Capturing multiday cycles requires long term monitoring and, in addition to demonstrated utility for forecasting, an understanding of seizure cycles may be critical for the development of new therapies.

We have also demonstrated the potential for seizure forecasting with sub-scalp systems. In this example, a forecaster achieved high accuracy (83%) and spent 26% of time in a high risk state, despite the high probability of seizures (2.2%) in this participant. These results are comparable to the only prospective seizure forecasting trial to-date, where patients spent 23% of their time in the warning state on average, but had a lower sensitivity of 66% (4). The AUC score (0.88) was also comparable to recent, state-of-the-art forecasting using interictal EA cycles derived from intracranial EEG (26, 32).

The case study demonstrates the high performance that can be achieved through an event-based seizure forecaster. This forecaster may be used to generate powerful prior probabilities for a more advanced seizure forecaster that combines other features, such as non-invasive information (e.g., medication adherence, heart rate etc.) and continuous features derived from the EEG (e.g., spectral power, autocorrelation etc.). Additionally, the forecast was able to continue making predictions despite the missing data during the period the device was not connected. Whilst cycles were attenuated during this period, seizure cycles were still utilized, as they rely on a fitted sinusoid of fixed period-length. The relative low likelihood of seizures during April compared to other months suggests that cycles in the detected events were stronger predictors of seizures than seizure cycles. This is in line with previous work, which suggests seizures are more robustly synchronized to cycles of a continuous biomarker than fitted sinusoids of fixed period-length (41).

Cyclic features in the EEG were stable and no adaptation period was required to start forecasting in this participant. The lack of implantation effect of sub-scalp systems (42) is in contrast to intracranial devices, which require a craniotomy and often a substantial time period before the signal stabilizes (21). This may require months of data to be discarded prior to training forecasting algorithms (28, 32). In this case, forecaster training was undertaken immediately, and only 14 days of training data were required to generate forecasts (although this will depend on seizure frequency). Further work will investigate the utility of forecasting using sub-scalp recordings in a prospective study.

There are limitations with sub-scalp EEG systems. First, despite the limited invasiveness of subcutaneous electrodes, this surgical procedure may not be acceptable to all people with epilepsy (43). Hence, patient seizure diaries will remain a useful tool in clinical settings, and non-invasive forecasting systems based on mobile and wearable devices are desired by the epilepsy community (43, 44). Wearable sensors and non-invasive features may be useful to forecast seizure likelihood (25, 45, 46), and self-reported events and biomarkers derived from wearables also demonstrate cycles that are co-modulated with seizure likelihood (30, 38, 40). However, the correlation between self-reported events and electrographic events is patient-specific. In cases where the accuracy is less than perfect, it is unlikely that forecasts using self-reported events will perform as well as forecasts using chronic EEG. Despite advances in wearable technology for seizure detection, there remain significant false positives and many seizure types are missed (47). It is likely that chronic sub-scalp EEG recordings will prove to be a critical “ground-truth” to develop wearable seizure detection and forecasting.

Second, validating electrographic seizures also remains a significant challenge, even with the aid of an algorithm detecting suspect events. A short 24-h segment of continuous EEG alone can take hours for a trained neurophysiologist to review, which is not viable for large scale use of sub-scalp devices, and so optimizing seizure detection algorithms will be critical. The time taken for clinical review placed several limitations on the validation of the signal quality and the algorithms used in this preliminary study. Qualitatively, EEG signals between scalp and sub-scalp were found to be similar (Figure 2). Furthermore, the algorithm presented in this work highlighted strong cycles in detected activity, which are similar to cycles of epileptiform activity observed in previous studies (8, 17, 18). However, a more comprehensive assessment of signal equivalence and algorithm performance is required and will be addressed in future work.

Third, the retrospective forecasting case study was only presented in one participant. We acknowledge that a larger cohort study is necessary to demonstrate the generalisability of our forecasting results. Finally, it should be noted that the highly clustered nature of the electrographic seizures in participant 1 may have aided the algorithm in achieving a high AUC score. On the other hand, clusters tend to result in short cycles, but the long 18d and 29d event cycles were the strongest predictors in this algorithm, and these are present irrespective of clusters. To understand this further, future work may investigate the forecasting performance on lead seizures only.

This study has demonstrated the feasibility of using a continuous sub-scalp EEG device to record data of sufficient resolution to capture relevant events, detect the events algorithmically, and use the events in a seizure forecasting algorithm. This data is extremely valuable for the assessment of epilepsy, and could be linked to systems to improve safety and independence, potentially changing fundamentally our approach to the management of the condition.

## Data Availability Statement

The datasets presented in this article are not readily available because data is commercially sensitive. Applications for reasonable use will be considered. Requests to access the datasets should be directed to Mark J. Cook, mark@seermedical.com.

## Ethics Statement

The studies involving human participants were reviewed and approved by St. Vincent's Hospital Melbourne Human Research Ethics Committee. The patients/participants provided their written informed consent to participate in this study.

## Disclosure

RS, MMa, PK, EN, KM, DF, and MC had employment or a financial interest in Seer Medical Pty. Ltd. MC, SR, JH, and RH have employment or a financial interest in Epi-Minder Pty. Ltd.

## Author Contributions

JH, SR, RH, AL, WD'S, US, LS, KM, KB, MMu, and MC contributed to the data collection. WD'S, US, LS, and KM contributed to the results analysis. SR, JH, RH, and AL, contributed to the manuscript writing. PK, EN, DG, BB, MR, DF, and MC contributed to the study and concept design and manuscript writing. RS and MMa contributed to the study and concept design, results analysis and manuscript writing. All authors contributed to the article and approved the submitted version.

## Conflict of Interest

RS, MMa, PK, EN, KM, DF, and MC were employed or have a financial interest in Seer Medical Pty. Ltd. MC, SR, JH, and RH were employed or have a financial interest in Epi-Minder Pty. Ltd. MR had a research collaboration with UNEEG medical and has been a member of their advisory board. BB had a financial interest in Cadence Neurosciences Inc., and had received nonfinancial research support (devices for a study) from Medtronic Inc. JH was employed by company Cochlear Limited. The remaining authors declare that the research was conducted in the absence of any commercial or financial relationships that could be construed as a potential conflict of interest. The handling editor VR declared a past co-authorship/collaboration with one or more authors PK and MC.

## Publisher's Note

All claims expressed in this article are solely those of the authors and do not necessarily represent those of their affiliated organizations, or those of the publisher, the editors and the reviewers. Any product that may be evaluated in this article, or claim that may be made by its manufacturer, is not guaranteed or endorsed by the publisher.

## References

[1] Reynolds JR. Epilepsy: its symptoms, treatment, and relation to other chronic, convulsive diseases. Am J Psychiatry. (1862) 19:198–209. 10.1176/ajp.19.2.198 30163555 PMC5180335

[2] Karoly P Goldenholz DM Cook M. Are the days of counting seizures numbered? Curr Opin Neurol. (2018) 31:162–8. 10.1097/WCO.0000000000000533 29369115

[3] Luoni C Bisulli F Canevini MP Sarro GD Fattore C Galimberti CA. Determinants of health-related quality of life in pharmacoresistant epilepsy: results from a large multicenter study of consecutively enrolled patients using validated quantitative assessments. Epilepsia. (2011) 52:2181–91. 10.1111/j.1528-1167.2011.03325.x 22136077

[4] Cook MJ O'Brien TJ Berkovic SF Murphy M Morokoff A Fabinyi G. Prediction of seizure likelihood with a long-term, implanted seizure advisory system in patients with drug-resistant epilepsy: a first-in-man study. Lancet Neurol. (2013) 12:563–71. 10.1016/S1474-4422(13)70075-9 23642342

[5] Loughman A Seneviratne U Bowden SC D'Souza WJ. Epilepsy beyond seizures: predicting enduring cognitive dysfunction in genetic generalized epilepsies. Epilepsy Behav. (2016) 62:297–303. 10.1016/j.yebeh.2016.07.010 27544704

[6] Ung H Cazares C Nanivadekar A Kini L Wagenaar J Becker D. Interictal epileptiform activity outside the seizure onset zone impacts cognition. Brain J Neurol. (2017) 140:2157–68. 10.1093/brain/awx143 28666338 PMC6167607

[7] Plummer C Vogrin SJ Woods WP Murphy MA Cook MJ Liley DTJ. Interictal and ictal source localization for epilepsy surgery using high-density EEG with MEG: a prospective long-term study. Brain. (2019) 142:932–51. 10.1093/brain/awz015 30805596 PMC6459284

[8] Karoly PJ Rao VR Gregg NM Worrell GA Bernard C Cook MJ. Cycles in epilepsy. Nat Rev Neurol. (2021) 17:267–84. 10.1038/s41582-021-00464-1 33723459

[9] Tatum WOI Winters L Gieron M Passaro EA Benbadis S Ferreira J. Outpatient seizure identification: results of 502 patients using computer-assisted ambulatory EEG. J Clin Neurophysiol. (2001) 18:14–9. 10.1097/00004691-200101000-00004 11290934

[10] Blum DE Eskola J Bortz JJ Fisher RS. Patient awareness of seizures. Neurology. (1996) 47:260–4. 10.1212/WNL.47.1.260 8710091

[11] Elger CE Hoppe C. Diagnostic challenges in epilepsy: seizure under-reporting and seizure detection. Lancet Neurol. (2018) 17:279–88. 10.1016/S1474-4422(18)30038-3 29452687

[12] Chen Z Brodie MJ Liew D Kwan P. Treatment outcomes in patients with newly diagnosed epilepsy treated with established and new antiepileptic drugs: a 30-year longitudinal cohort study. JAMA Neurol. (2018) 75:279–86. 10.1001/jamaneurol.2017.3949 29279892 PMC5885858

[13] Gliske SV Irwin ZT Chestek C Hegeman GL Brinkmann B Sagher O. Variability in the location of high frequency oscillations during prolonged intracranial EEG recordings. Nat Commun. (2018) 9:1–14. 10.1038/s41467-018-04549-2 29858570 PMC5984620

[14] Chen Z Grayden DB Burkitt AN Seneviratne U D'Souza WJ French C. Spatiotemporal patterns of high-frequency activity (80-170 hz) in long-term intracranial eEG. Neurology. (2020) 96. 10.1101/2020.03.26.999425 33361261

[15] Goldenholz DM Moss R Scott J Auh S Theodore WH. Confusing placebo effect with natural history in epilepsy: a big data approach. Ann Neurol. (2015) 78:329–36. 10.1002/ana.24470 26150090 PMC4546516

[16] Karoly PJ Romero J Cook MJ Freestone DR Goldenholz DM. When can we trust responders? Serious concerns when using 50% response rate to assess clinical trials. Epilepsia. (2019) 60:e99–103. 10.1111/epi.16321 31471901

[17] Baud MO Kleen JK Mirro EA Andrechak JC King-Stephens D Chang EF. Multi-day rhythms modulate seizure risk in epilepsy. Nat Commun. (2018) 9:88. 10.1038/s41467-017-02577-y 29311566 PMC5758806

[18] Karoly PJ Freestone DR Boston R Grayden DB Himes D Leyde K. Interictal spikes and epileptic seizures: their relationship and underlying rhythmicity. Brain. (2016) 139:1066–78. 10.1093/brain/aww019 26912639

[19] Duun-Henriksen J Baud M Richardson MP Cook M Kouvas G Heasman JM. A new era in electroencephalographic monitoring? Subscalp devices for ultra–long-term recordings. Epilepsia. (2020) 61:1805–17. 10.1111/epi.16630 32852091

[20] Viana PF Duun-Henriksen J Glasstëter M Dümpelmann M Nurse ES Martins IP. 230 days of ultra long-term subcutaneous EEG: seizure cycle analysis and comparison to patient diary. Ann Clin Transl Neurol. (2021) 8:288–93. 10.1002/acn3.51261 33275838 PMC7818131

[21] Ung H Baldassano SN Bink H Krieger AM Williams S Vitale F. Intracranial eEG fluctuates over months after implanting electrodes in human brain. J Neural Eng. (2017) 14:056011. 10.1088/1741-2552/aa7f40 28862995 PMC5860812

[22] Nurse ES John SE Freestone DR Oxley TJ Ung H Berkovic SF. Consistency of long-term subdural electrocorticography in humans. IEEE Trans Biomed Eng. (2018) 65:344–52. 10.1109/TBME.2017.2768442 29364119

[23] Spatiotemporal Patterns of High-Frequency Activity (80–170 Hz) in Long-Term Intracranial EEG | Neurology. Available online at: https://n.neurology.org/content/96/7/e1070.long (accessed April 22, 2021). 10.1212/WNL.0000000000011408 33361261

[24] Dell KL Cook MJ Maturana MI. Deep brain stimulation for epilepsy: biomarkers for optimization. Curr Treat Options Neurol. (2019) 21:47. 10.1007/s11940-019-0590-1 31559493

[25] Payne DE Dell KL Karoly PJ Kremen V Gerla V Kuhlmann L. Identifying seizure risk factors: a comparison of sleep, weather, and temporal features using a bayesian forecast. Epilepsia. (2021) 62:371–382. 10.1111/epi.16785 33377501 PMC8012030

[26] Maturana MI Meisel C Dell K Karoly PJ D'Souza W Grayden DB. Critical slowing down as a biomarker for seizure susceptibility. Nat Commun. (2020) 11:2172. 10.1038/s41467-020-15908-3 32358560 PMC7195436

[27] Kuhlmann L Lehnertz K Richardson MP Schelter B Zaveri HP. Seizure prediction — ready for a new era. Nat Rev Neurol. (2018) 14:618–630. 10.1038/s41582-018-0055-2 30131521

[28] Karoly PJ Ung H Grayden DB Kuhlmann L Leyde K Cook MJ. The circadian profile of epilepsy improves seizure forecasting. Brain. (2017) 140:2169–82. 10.1093/brain/awx173 28899023

[29] Karoly PJ Goldenholz DM Freestone DR Moss RE Grayden DB Theodore WH. Circadian and circaseptan rhythms in human epilepsy: a retrospective cohort study. Lancet Neurol. (2018) 17:977–85. 10.1016/S1474-4422(18)30274-6 30219655

[30] Karoly PJ Cook MJ Maturana M Nurse ES Payne D Brinkmann BH. Forecasting cycles of seizure likelihood. Epilepsia. (2020) 61:776–86. 10.1101/2019.12.19.19015453 32219856

[31] Leguia MG Andrzejak RG Rummel C Fan JM Mirro EA Tcheng TK. Seizure cycles in focal epilepsy. JAMA Neurol. (2021) 78:454–63. 10.1001/jamaneurol.2020.5370 33555292 PMC7871210

[32] Proix T Truccolo W Leguia MG Tcheng TK King-Stephens D Rao VR. Forecasting seizure risk in adults with focal epilepsy: a development and validation study. Lancet Neurol. (2021) 20:127–35. 10.1016/S1474-4422(20)30396-3 33341149 PMC7968722

[33] Scheffer IE Berkovic S Capovilla G Connolly MB French J Guilhoto L. ILAE classification of the epilepsies: position paper of the ILAE commission for classification and terminology. Epilepsia. (2017) 58:512–21. 10.1111/epi.13709 28276062 PMC5386840

[34] Clarke S Karoly PJ Nurse E Seneviratne U Taylor J Knight-Sadler R. Computer-assisted EEG diagnostic review for idiopathic generalized epilepsy. Epilepsy Behav. (2019) 106556. 10.1101/682112 31676240

[35] Eden D Nurse ES Clarke S Karoly PJ Seneviratne U Cook M. Computer-assisted estimation of interictal discharge burden in idiopathic generalized epilepsy. Epilepsy Behav EB. (2020) 105:106970. 10.1016/j.yebeh.2020.106970 32114187

[36] Torrence C Compo GP. A practical guide to wavelet analysis. Bull Am Meteorol Soc. (1998) 79:61–78. 10.1175/1520-0477(1998)079&lt;0061:APGTWA&gt;2.0.CO;2

[37] Berens P. CircStat: a MATLAB toolbox for circular statistics. J Stat Softw. (2009) 37:1–21. 10.18637/jss.v031.i10

[38] Karoly PJ Eden D Nurse ES Cook MJ Taylor J Dumanis S. Cycles of self-reported seizure likelihood correspond to yield of diagnostic epilepsy monitoring. Epilepsia. (2021) 62:416–25. 10.1111/epi.16809 33507573

[39] Dell KL Payne DE Kremen V Maturana MI Gerla V Nejedly P. Seizure likelihood varies with day-to-day variations in sleep duration in patients with refractory focal epilepsy: a longitudinal electroencephalography investigation. EClin Med. (2021). 37:100934. 10.1016/j.eclinm.2021.100934 PMC8343264 34386736

[40] Karoly PJ Stirling RE Freestone DR Nurse ES Doyle B Halliday A. Multiday cycles of heart rate modulate seizure likelihood at daily, weekly and monthly timescales: an observational cohort study. medRxiv. (2020). 10.1101/2020.11.24.20237990

[41] Leguia MG Rao VR Kleen JK Baud MO. Measuring synchrony in bio-medical timeseries. Chaos Interdiscip J Nonlinear Sci. (2021) 31:013138. 10.1063/5.0026733 33754758

[42] Viana PF Remvig LS Duun-Henriksen J Glasstetter M Dümpelmann M Nurse ES. Signal quality and power spectrum analysis of remote ultra long-term subcutaneous EEG. Epilepsia. (2021) 1–9. 10.1111/epi.16969 34250608

[43] Janse SA Dumanis SB Huwig T Hyman S Fureman BE Bridges JFP. Patient and caregiver preferences for the potential benefits and risks of a seizure forecasting device: a best–worst scaling. Epilepsy Behav. (2019) 96:183–91. 10.1016/j.yebeh.2019.04.018 31150998

[44] Dumanis SB French JA Bernard C Worrell GA Fureman BE. Seizure forecasting from idea to reality. Outcomes of the My Seizure Gauge Epilepsy Innovation Institute Workshop. Eneuro. (2017) 4:ENEURO.0349-17.2017. 10.1523/ENEURO.0349-17.2017 29291239 PMC5744646

[45] Meisel C El Atrache R Jackson M Schubach S Ufongene C Loddenkemper T. Machine learning from wristband sensor data for wearable, noninvasive seizure forecasting. Epilepsia. (2020) 61:2653–66. 10.1111/epi.16719 33040327

[46] Stirling RE Grayden DB D'Souza W Cook MJ Nurse E Freestone DR. Forecasting seizure likelihood with wearable technology. Front Neurol. (2021) 12:704060. 10.3389/fneur.2021.704060 34335457 PMC8320020

[47] Beniczky S Karoly P Nurse E Ryvlin P Cook M. Machine learning and wearable devices of the future. Epilepsia. (2020) 62:116–24. 10.1111/epi.16555 32712958

